# Study on the Dissolution Mechanism of Cellulose by ChCl-Based Deep Eutectic Solvents

**DOI:** 10.3390/ma13020278

**Published:** 2020-01-08

**Authors:** Heng Zhang, Jinyan Lang, Ping Lan, Hongyan Yang, Junliang Lu, Zhe Wang

**Affiliations:** 1 College of Marine Science and Biological Engineering, Qingdao University of Science & Technology, Qingdao 266042, China; ljy17806248212@163.com (J.L.); kdjh401@163.com (H.Y.); juling_lu@163.com (J.L.); 17853246018@163.com (Z.W.); 2 Guangxi Key Laboratory of Polysaccharide Materials and Modification, School of Chemistry and Chemical Engineering, Guangxi University for Nationalities, Nanning 530008, China; gxLanping@163.com; 3 Key Laboratory of Biomass Chemical Engineering of Ministry of Education, Zhejiang University, Hangzhou 310027, China

**Keywords:** Deep eutectic solvent (DES), cellulose, dissolution, hydrogen bond, ChCl

## Abstract

Four deep eutectic solvents (DESs), namely, glycerol/chlorocholine (glycerol/ChCl), urea/ChCl, citric acid/ChCl, and oxalic acid/ChCl, were synthesized and their performance in the dissolution of cellulose was studied. The results showed that the melting point of the DESs varied with the proportion of the hydrogen bond donor material. The viscosity of the DESs changed considerably with the change in temperature; as the temperature increased, the viscosity decreased and the electrical conductivity increased. Oxalic acid/ChCl exhibited the best dissolution effects on cellulose. The microscopic morphology of cellulose was observed with a microscope. The solvent system effectively dissolved the cellulose, and the dissolution method of the oxalic acid/ChCl solvent on cellulose was preliminarily analyzed. The ChCl solvent formed new hydrogen bonds with the hydroxyl groups of the cellulose through its oxygen atom in the hydroxyl group and its nitrogen atom in the amino group. That is to say, after the deep eutectic melt formed an internal hydrogen bond, a large number of remaining ions formed a hydrogen bond with the hydroxyl groups of the cellulose, resulting in a great dissolution of the cellulose. Although the cellulose and regenerated cellulose had similar structures, the crystal form of cellulose changed from type I to type II.

## 1. Introduction

Cellulose is a natural, abundant, and renewable macromolecular resource [1] that can be converted into various materials, fuels, and chemicals needed by humans. Hydrolysis, hydrogenation, pyrolysis, dehydration, and other reactions can effectively convert cellulose into glucose, sorbitol, ethylene glycol, synthetic gas, aromatic hydrocarbons, and furan compounds that are considered the future of biorefining “building blocks” [2,3]. In view of the existence of intermolecular and intramolecular hydrogen bonds in cellulose, the formation of highly crystalline supramolecular structures hinders its dissolution. Traditional techniques of dissolving cellulose include the use of copper ammonia, viscose solvents, inorganic acid solvents [4] and non-aqueous system solvents [5]. However, these solvents are unstable, toxic, and difficult to recycle, and therefore fail to satisfy the development requirements of the green chemical industry [6]. In the solubilization-catalyzed conversion of cellulose, an ionic liquid is widely used due to its excellent solubility in cellulose [7]. Although ionic liquids have shown superior properties in dissolving cellulose, the preparation of ionic liquids is complex and costly, which limits its industrial production. Additionally, many reports have pointed out that many ionic liquids are toxic and not easily biodegradable [8], which does not conform to the concept of sustainable development. Therefore, the development of new solvents for cellulose has always been a popular topic worldwide.

Abbott [9] et al. found that chlorocholine (ChCl) and urea can form a homogeneous solution at normal temperatures and proposed the concept of deep eutectic solvents (DESs), that is, combining a hydrogen bond acceptor (HBA) and a hydrogen bond donor (HBD) via hydrogen bonds at a certain molar ratio. The melting point of the eutectic mixture is lower than that of either of the single components. This solvent has many similar physical and chemical properties to those of ionic liquids. As such, DESs are also considered to be ionic liquids. Compared with other ionic liquids, DESs present the advantages of lower cost, simpler preparation, and greater biodegradability, safety, and an innocuous nature. Thus, they can truly realize the potential of green chemistry. Xing [10] et al. reported that cellulose can be dissolved in the DES of glycerol/K_2_CO_3_. Liu [11] et al. studied amidic-based DESs synthesized by solid organic compounds and preliminarily analyzed the mechanism of the dissolution of cellulose in the amidic-based DESs. Chlorocholoine is the most commonly used HBA because it is low cost, biodegradable, safe, non-toxic, and extractable from biomass energy. Chlorocholine can be combined with urea, glycerol, polyol, shuttle acid, and other safe and low-cost HBDs to form DESs. Ren [12] et al. synthesized four kinds of ChCl-based DESs and investigated the effects of their physical properties on the dissolution of cellulose. At present, deep eutectic solvent is only considered as a good solvent for lignin [13,14] and monosaccharide [15,16]. Research on choline chloride as a deep eutectic solvent is relatively sparse, and research on cellulose dissolution by choline chloride deep eutectic solvent is lacking. Cellulose is the most abundant renewable biomass resource on the earth [17]. Degradation of cellulose can obtain high-value chemical intermediates such as 5-hydroxymethylfurfural [18,19] and levulinic acid [20,21]. Therefore, it is particularly important to find a green and economic solvent that can effectively dissolve cellulose. However, as mentioned above, limited research has explored the dissolution of cellulose by ChCl-based DESs.

This experiment used glycerol, oxalic acid, citric acid, urea, and ChCl to synthesize a series of DESs for dissolving cellulose. The effects of various physical properties, such as the melting point, viscosity, and conductivity, of the DESs on the dissolution of cellulose and the properties of the resulting dissolved cellulose were investigated. The dissolution mechanism of cellulose by ChCl-based DESs was preliminarily explained. Given the lack of systematic investigations on cellulose systems dissolved by DESs, this study could provide a new way to design suitable solvents for cellulose.

## 2. Materials and Methods

### 2.1. Materials and Equipment

Glycerol, oxalic acid, citric acid, urea, and ChCl were the analytical reagents used and were provided by Sinopharm Chemical Reagent Co., Ltd.; microcrystalline cellulose (pharmaceutical grade) was provided by Chron Chemicals Co., Ltd. (Shanghai, China).

A SGO-PH201-type microscope was purchased from Fortune Technology Co., Ltd. (Shenzhen, China). The DDS-11A-type conductivity meter was provided by Shanghai INESA Scientific Instrument Co., Ltd. (Shanghai, China). Rotary rheometers were conducted using a MCR301 by Germany Anton Paar Co., Ltd. (Shanghai, China). X-ray diffraction (XRD) was conducted using a DX-2700 by Brook Spectrum Instrument Co., Ltd. (Ettlingen, Germany). A T27-type Fourier-transfer infrared spectroscopy (FT-IR; Resolution: 4 cm^−1^; Number of scans: 16) infrared instrument was provided by Bruker Spectrum Instrument Co., Ltd. (Bruker, Germany).

### 2.2. Experimental Method

#### 2.2.1. Synthesis of ChCl-DES’s

The one-step synthesis method [22] was used to synthesize the DESs. The reagents were dried in a vacuum oven at 50 °C for 48 h and then added to a four-necked flask at a certain molar ratio. The oil bath was heated at a certain temperature and nitrogen gas was applied to protect it during heating. After the sample turned into a transparent liquid, it was cooled at room temperature, dried in a vacuum drying oven at 70 °C for 48 h, transferred to a desiccator, and sealed.

#### 2.2.2. Physical Properties

The 100 g of DESs was weighed, dried, and heated in an oven at 105 °C to determine the moisture content.

The 100 g of DESs was added to the rheometer test platform. The temperature was increased from room temperature to 100 °C at a rate of 10 °C/min. The trace amount of moisture that may have remained in the DESs was removed at a constant temperature for 30 min. The temperature was reduced to 20 °C and maintained for 10 min. The temperature was further increased to 80 °C at 20 °C/min.

The 100 g of DESs was weighed in a flask and placed in a constant-temperature water bath, heated to a set temperature at a constant rate, collected, and immediately measured for the conductivity of the solvent at different temperatures with a conductivity meter.

#### 2.2.3. Dissolution of Cellulose

The prepared DES was added with cellulose in the four-necked flask, which was placed in an oil bath at the specified temperature. After the reaction was completed, the material was separated by filtration, and the residue was washed with distilled water, dried at 75 °C and weighed. The dissolved cellulose mass fraction was calculated using Equation (1),

(1) $$X=\frac{M-M_{C}}{M}\times 100$$

where X is the cellulose dissolution rate (%) and *M* and *M_C_* are the mass (g) and dryness (g) of the cellulose, respectively.

#### 2.2.4. Performance Testing

A SEM microscope was used to observe the morphological characteristics of cellulose before, during, and after dissolution. The DX-2700 model X-ray diffractometer was used to characterize the samples under 2θ of 5° to 50° and the change in the crystal shape was analyzed. The samples were compressed using KBr and the DES’s infrared spectra were obtained using an FT-IR infrared instrument.

## 3. Results and Discussion

### 3.1. Physical Properties of DESs and the Solubility of Cellulose

Similar to that of ionic liquids, the melting point of DESs depends on the melting point of the starting compound and the interaction between the anions and cations. The bond energy of the quaternary ammonium salt and the HBD, as well as the entropy change before and after the formation of the liquid, determine the melting point of the prepared DESs [23].

The melting points of HBA, HBD and DESs respectively represent the melting points of hydrogen bond acceptors, hydrogen bond donors and deep eutectic solvents.

It can be seen from Table 1 that the deep eutectic ratio is choline chloride: hydrogen bond donor = 1:2 (molar ratio). The melting temperatures of glycerol/choline chloride, urea/choline chloride, citric acid/choline chloride and oxalic acid/choline chloride were −36.5 °C, 65 °C, 37.8 °C and 30 °C, respectively. Therefore, the melting point of deep eutectic solvent is lower than that of any single component under the effect of hydrogen bonds. This occurs because when the two molecules were heated and mixed, the intramolecular hydrogen bonds were destroyed and new intermolecular hydrogen bonds were formed between choline chloride and HBD molecules. The hydrogen bond energy was reduced, the molecular arrangement was irregular and the crystallinity was reduced, so the freezing point of the new system was lowered.

As shown in Table 1, the solubilities of glycerol/ChCl, urea/ChCl, citric acid/ChCl, and oxalic acid/ChCl were 0.6%, 1.03%, 1.94%, and 2.54%, respectively. This wide liquid range at room temperature corresponded to a large number of stable properties. Glycerol was in liquid form at room temperature and the DESs formed with ChCl had a low melting point. A homogeneous solvent was easily formed without heating and existed in liquid form at room temperature and had a wide range of applications. The eutectic solvent formed by ChCl and oxalic acid had a melting point of 30 °C and displayed fluidity at room temperature. The two deep solvents had wide ranges of temperatures in which they both remained in liquid form and were stable at the same time. In summary, the combination of ChCl and the HBDs greatly reduced the melting point of the single components, leading to liquid systems with low temperatures, which are favorable conditions for chemical reactions.

### 3.2. Effect of Temperature on the Viscosity of DESs

Viscosity is an important property of DESs. Hydrogen bonding forces, the Van der Waals force, and electrostatic forces are the main affecting factors of the viscosity of solvents. From a macroscopic perspective, dense and powerful hydrogen bonding forces among compounds hinder the movement of free molecules, leading to the high viscosity of DESs. From a microcosmic perspective, small free volumes among the solvent molecules lead to large ionic entities of the solvent and allow the electrostatic and Van der Waals forces among the molecules to promote the formation of high-viscosity DESs. A low viscosity is beneficial to the mass transfer between a solute and a solvent.

**Figure 1:**
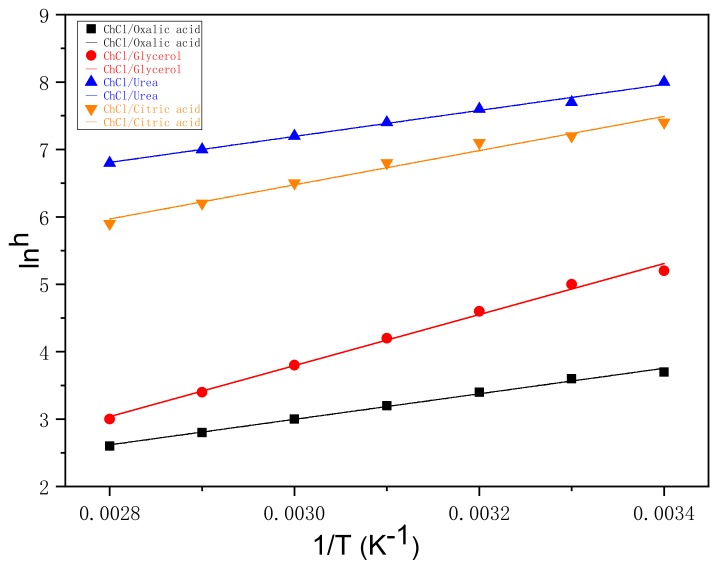
The effect of temperature on the viscosity of DESs.

Figure 1 shows the viscosity of four different DESs, namely, glycerol/ChCl, urea/ChCl, citric acid/ChCl, and oxalic acid/ChCl. The test range was from 20 °C to 80 °C.

As shown in Figure 1, the viscosity decreased with increased temperature. The viscosities of urea/ChCl and citric acid/ChCl were clearly higher than those of the two other DESs. Glycerol/ChCl was the secondl, and oxalic acid/ChCl had the lowest viscosity. As the temperature increased to 50 °C, the viscosity of the DESs gradually changed because the sensitivity of the molecules of the DESs to temperatures beyond 50 °C decreased. As the temperature increased, the viscosity of the solvent gradually decreased because of the gradual weakening of the Van der Waals and hydrogen bonding forces during the heating process. An adequate kinetic energy was obtained to overcome these internal forces and a large number of hydrogen bonds were formed, which destroyed the crystal arrangement of the original molecules and formed a new supramolecular system. Consequently, the molecular internal force of the original solid was weakened, enabling it to move more freely. Low viscosity is beneficial to the mass transfer between a solute and a solvent, as it increases the probability of intermolecular collisions. A small viscosity corresponds to a high permeability of the tightly structured cellulose, which is beneficial to the formation of new molecular hydrogen bonds between DESs and cellulose, thereby increasing the solubility of cellulose.

### 3.3. Effect of Temperature on the Electrical Conductivity of DESs

**Figure 2:**
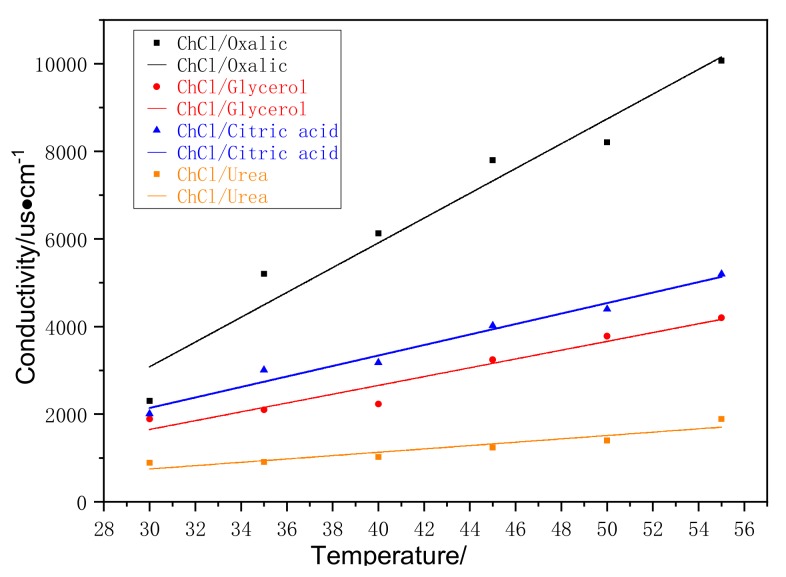
The effect of temperature on the conductivity of DES’s.

Figure 2 shows the conductivities of four different DESs, namely, glycerol/ChCl, urea/ChCl, citric acid/ChCl, and oxalic acid/ChCl. The test range was from 30 °C to 55 °C.

Electrical conductivity is generally used to characterize the ability of materials to transport current, which is related to the movement of charged particles and the vibration of free particles [24]. Therefore, the quantity and the mobility of the charged particles affects the electrical conductivity. The low-temperature eutectic solvent prepared in this experiment had a low electrical conductivity, which indicated that there was a relatively small amount of freely moving charged particles in the system. For this system, the moving particles were not individual ions but supramolecular and free molecules. Supramolecular migration leads to the formation of large supramolecular molecules. In this process, supramolecular molecules can decompose into smaller supramolecular molecules. Therefore, electrical conductivity is dependent not on the ions but on the movement of the supramolecular and free molecules. As such, the DESs had low electrical conductivities [11].

As shown in Figure 2, the electrical conductivity increased with increased temperature. As temperature increased, the viscosity of the system was reduced; the molecules had been heated to produce sufficient kinetic energy to accelerate the movement of the anions and the cations, increasing the probability of collisions among molecules, weakening the interactions among molecules, and increasing the electrical conductivity of the system. Figure 2 also shows that the relationship between the electrical conductivity and temperature of the DESs was opposite to the viscosity-temperature relationship and that the low-viscosity DESs had a high conductivity. In terms of the relationship between electrical conductivity and temperature, the DESs could be ordered as follows: oxalic acid/ChCl > citric acid/ChCl > glycerol/ChCl > urea/ChCl. This sequence was consistent with that of the relationship between solubility and temperature; the viscosity of urea choline was obviously higher than that of other DESs and its electrical conductivity was the smallest. Moreover, compared to glycerol/ChCl and citric acid/ChCl with a multi hydroxyl HBD, oxalic acid/ChCl with a binary hydroxyl HBD had a relatively high electrical conductivity. When fewer hydroxyl groups existed in the system, the hydrogen bond network was more loosely formed and thus was beneficial for the activity of molecules and the contact between the DESs and cellulose molecules [25]. These findings confirmed that the structure, size, and properties of particles strongly affected the electrical conductivity. A high electrical conductivity led to the violent movement of molecules, increasing the collision probability between molecules. This phenomenon was conducive to the dissolution of cellulose.

### 3.4. Effect of Temperature on Cellulose Dissolution

Temperature is a key influencing factor of the solubility of lignin in DESs. The effect of temperature on the cellulose dissolution rate of DESs is shown in Table 2.

As shown in Table 2, temperature greatly influenced the dissolution rate of cellulose in the DESs. As the temperature increased, the solubility increased. The reason for this trend was that spontaneous dissolution of cellulose occurred when the change in the Gibbs free energy of the mixture, ΔG_M_, was less than zero. This is further described by Equation (2),

ΔG_M_ = ΔH_M_ − TΔS_M_ < 0 (2)

where ΔG_M_ (KJ/mol) is the mixed heat, ΔS_M_ (KJ/K·mol) is the mixed entropy, and T (K) is the dissolution temperature.

When the cellulose was dissolved, the molecular arrangement became turbulent and the entropy of the system was increased, suggesting that ΔS_M_ > 0. The dissolution was an endothermic process where ΔH_M_ (KJ/mol) > 0. When |ΔH_M_| < |TΔS_M_|, ΔG_M_ < 0 holds. Therefore, the dissolution of cellulose in the DESs was promoted and the solubility of cellulose increased when the temperature increased [26]. When the temperature was lower than 80 °C, the dissolution rate of cellulose increased slowly with increases in temperature. The reason was that the degree of ions ionization decreased at low temperatures, which was not conducive to molecular activities and led to poor electrical conductivity and a high solvent viscosity. The high viscosity was not conducive to mass transfer between the solute and the solvent. Thus, the chance of intermolecular collisions was reduced, making it difficult for the DESs to form new molecular hydrogen bonds with cellulose. When the temperature was higher than 80 °C, both the dissolution equilibrium constant and the catalytic transfer ability of the quaternary ammonium salt increased as the temperature increased, resulting in the removal of more cellulose. Thus, the saturated solubility of cellulose in DESs was gradually increased. As the temperature continued to increase, the dissolution rate of cellulose did not increase considerably, which suggested that the cellulose dissolution rate approached or reached saturation in the DESs.

In summary, DESs had many physical and chemical properties that were similar to those of ionic liquids, but the solubility of their cellulose varied. The solubility of cellulose in ionic liquids was approximately 15%, whereas that in DESs reached only up to nearly 2%.

### 3.5. Observation of SEM during Cellulose Dissolution

**Figure 3:**
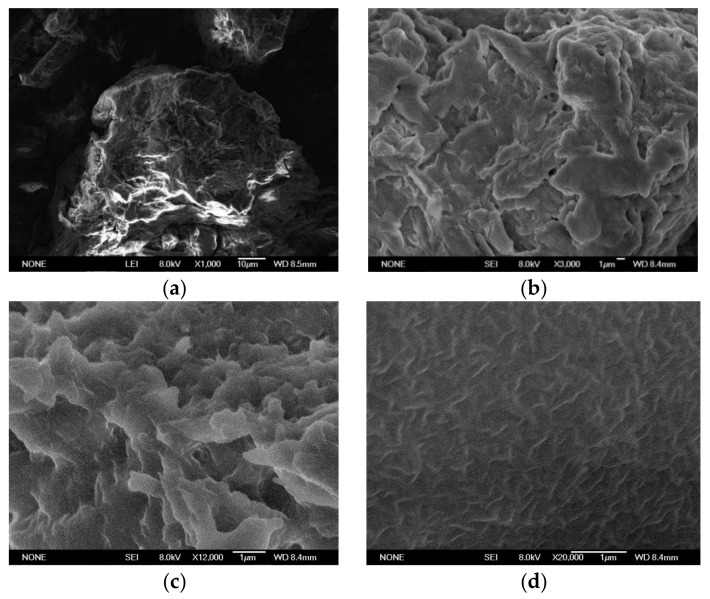
Dissolution of cellulose in oxalic acid-chlorocholine. DES: (**a**) The SEM of cellulose before dissolving; (**b**) cellulose SEM after reaction 30 min; (**c**) cellulose SEM after reaction 60 min; (**d**) cellulose SEM after reaction 90 min.

Figure 3 shows the micromorphology of cellulose dissolved in a urea/ChCl eutectic system at 100 °C. Visibly, the basic form of cellulose was changed by dissolution. The pre-dissolved cellulose flocculated into a group, and the structure was relatively tight. The cellulose in the oxalic acid/ChCl deep eutectic system showed a deterioration within a short time, and the cellulose became considerably short, thin, and small, and ultimately disappeared. These results proved that the cellulose dissolution was very clear in this system. Moreover, the dissolution rate was very fast, and the cellulose was completely dissolved after 2 h.

### 3.6. Determination of Regenerated Cellulose by XRD

**Figure 4:**
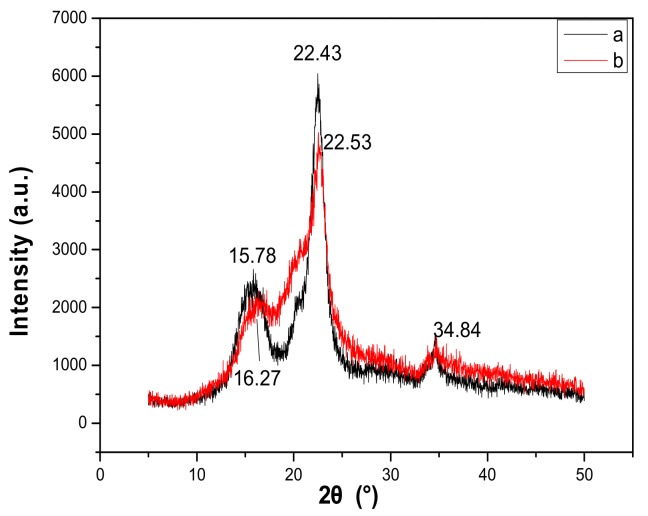
XRD spectra of microcrystalline cellulose (**a**) and cellulose dissolved in oxalic acid chlorocholine DES (**b**).

The results obtained by X-ray diffraction (Figure 4) showed that the diffraction spectra of the cellulose regenerated from the oxalic acid/ChCl solvent system was similar to that of the original cellulose. No other by-products were formed during the dissolution of cellulose, proving the feasibility of using oxalic acid/ChCl. The characteristic peaks of the cellulose I crystal form appeared at 15.78°, 22.43°, and 34.8° [27]. After cellulose was regenerated, the XRD spectra showed a broad crystalline peak at 16.27° to 22.53°, which indicated that the cellulose structure changed to a typical cellulose II structure. These results implied that the dissolution of cellulose by the solvent was a physical change. In the process of dissolving cellulose, only the crystallinity of the cellulose was reduced, and the chemical properties were unchanged.

### 3.7. Infrared Spectral Analysis

**Figure 5:**
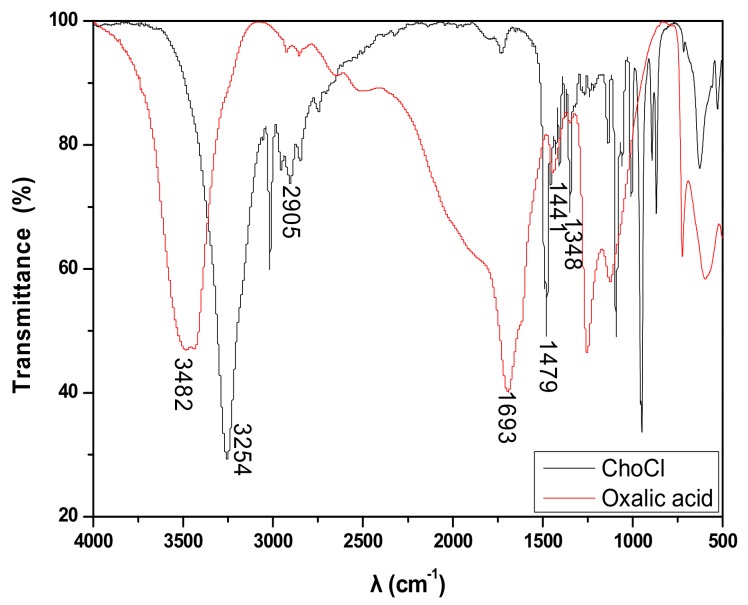
The IR spectrum of choline chloride and oxalic acid.

**Figure 6:**
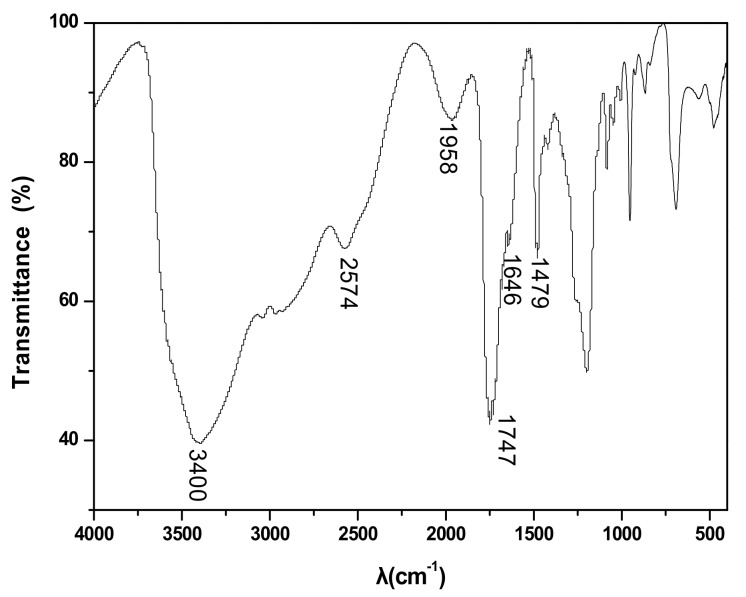
The IR spectrum of choline chloride-oxalic acid.

Figure 5 shows that the C–H vibration peak of ChCl appeared at 2905.82 cm^−1^ and that the characteristic peak of halogenated organic compounds appeared at 1734.16 cm^−1^. The infrared absorption peak of the C–N stretching vibration appeared at 1348.26 cm^−1^, and the C=O vibrational absorption peak of oxalic acid was near 1693.67 cm^−1^. The O–H vibrational absorption peaks were near 1441.55 cm^−1^ and 1252.79 cm^−1^. A comparison of Figure 5 and Figure 6 revealed that the infrared absorption peak of the C–N stretching vibration of ChCl disappeared, signifying the movement of the C=O vibration absorption peak of oxalic acid toward a higher wave number direction (from 1693.67 cm^−1^ to 1747.56 cm^−1^ and 1958.85 cm^−1^) due to the strong electronegativity of Cl^−^ and the induction effect [28]. The peak band narrowed and weakened, and the vibrational absorption peak of O–H disappeared, which implied that the intramolecular hydrogen bonds of ChCl and oxalic acid disappeared. The characteristic peak of the oxalic acid/ChCl DES at 2574.78 cm^−1^ was an intramolecular hydrogen bond, thereby confirming the formation of new hydrogen bonds in the solvent.

### 3.8. Dissolution Mechanism of Cellulose in DESs

**Figure 7:**
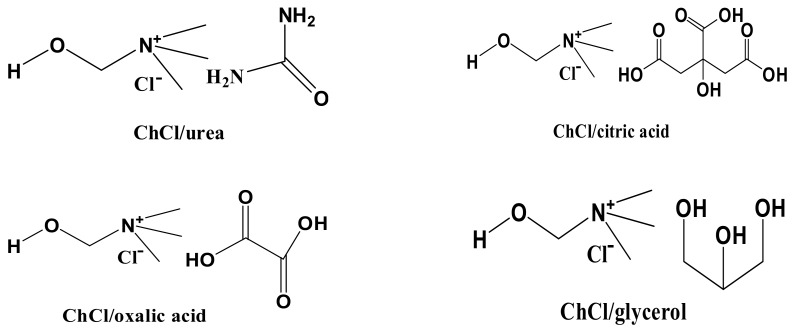
The structure of the four choline-chloride-based DESs.

**Figure 8:**
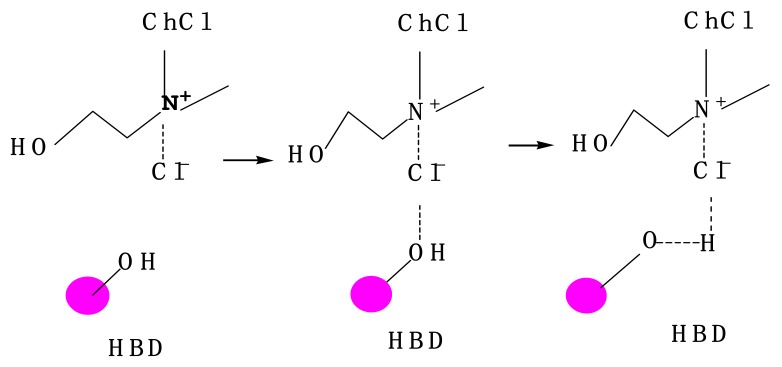
Synthetic route of ChCl/HBD [29].

As shown in Table 1, the four solvents could somewhat dissolve cellulose but at low levels. The order of solvency was oxalic acid/ChCl > citric acid/ChCl > urea/ChCl > glycerol/ChCl. The DESs did not weaken the interactions between anions and cations in hydrogen bonds in cellulose but did compete with the cellulose hydrogen bonds [11]. The DESs, in addition to the internal hydrogen bonds, also formed hydrogen bonds with cellulose and dissolved cellulose. Therefore, analyzing the hydrogen bond formation ability and the hydrogen bond type is critical in understanding the dissolution of cellulose. One amine group can form three hydrogen bonds (two hydrogen atoms on the nitrogen atom and a lone pair of nitrogen atoms). One carbonyl oxygen can form two hydrogen bonds, and one hydroxyl oxygen can form one hydrogen bond. As shown in Figure 7, one urea molecule, which has one carbonyl group and two amine groups, can form eight hydrogen bonds. Similarly, one oxalic acid molecule, which has two hydroxyl groups and two carbonyl groups, can form six hydrogen bonds. It follows that one glycerol molecule (and its three hydroxyl groups) can form three hydrogen bonds and one citric acid molecule (which has four hydroxyl groups and three carbonyl groups) can form 10 hydrogen bonds. The ChCl can accept three H+. As depicted in Figure 8, three hydrogen bonds can be formed between urea and ChCl. The carbonyl oxygen atom of urea can also form a hydrogen bond. The hydroxyl group can form additional hydrogen bonds with cellulose and the other amine group of urea can form three hydrogen bonds with cellulose. Thus, urea and cellulose can form a total of four hydrogen bonds. Similarly, oxalic acid and ChCl can form two hydrogen bonds. The ChCl can form a hydrogen bond with the hydroxyl group of cellulose, and oxalic acid molecules can form six hydrogen bonds with cellulose, giving a total of seven bonds with cellulose. Citric acid can form three hydrogen bonds with ChCl and seven hydrogen bonds with cellulose. Glycerol can form three hydrogen bonds with ChCl and three hydrogen bonds with cellulose. Given that the electronegativity of oxygen is greater than that of nitrogen, the hydrogen bonds formed by the amine nitrogen atoms were not as strong as those formed by the carbonyl oxygen. Thus, the solubility of cellulose in oxalic acid/ChCl and citric acid/ChCl was higher than that of the two other DESs. Although the viscosity of glycerol/ChCl was low, the number of hydrogen bonds with cellulose was still relatively small, causing cellulose’s solubility in that solution to be relatively low.

Molecular chains inside cellulose are aggregated into a highly ordered network structure due to the extensive presence of hydrogen bonds. This strong hydrogen bonding network hinders the dissolution of cellulose in conventional solvents, such as water and most organic solvents [30]. However, the solubility of cellulose in DESs depends on the number of hydrogen bonds formed by free ions and cellulose and the strength of those hydrogen bonds, or the total bond energy that forms hydrogen bonds with the hydroxyl groups in the cellulose chain. A large total bond energy corresponded to a high solubility of cellulose. After the deep eutectic system formed internal hydrogen bonds, the ability of the remaining ions to form hydrogen bonds with the hydroxyl groups of cellulose became strong and the solubility of the cellulose became high. The mechanism by which the ionic liquid dissolved cellulose is that the high concentration of Cl^−^ in the ionic liquid enhanced the ability of the solvent to destroy the hydrogen bonds of the cellulose. Swatloski [31] posited that Cl^−^-containing ionic liquids can dissolve cellulose, whereas ionic liquids containing anionic BF-4 or PF-6 cannot dissolve cellulose. The probable reason for this is that Cl^−^ can form hydrogen bonds with the hydroxyl groups in the cellulose molecular chains, thereby weakening the intramolecular or intermolecular hydrogen bonds of cellulose. Moulthrop [32] et al. verified this mechanism by conducting experiments. The preceding analysis revealed that the Cl^−^ in ionic liquids directly forms hydrogen bonds with the hydroxyl groups of cellulose and does not need to form internal hydrogen bonds with itself. After the deep eutectic system formed internal hydrogen bonds, the remaining electron-reactive ions became able to be transported towards the hydroxyl groups of the cellulose and form hydrogen bonds, explaining the greater solubility of cellulose in ionic liquids than in DESs.

## 4. Conclusions

Oxalic acid/ChCl, citric acid/ChCl, urea/ChCl, and glycerol/ChCl were manufactured and used to determine the effects of viscosity, conductivity, and temperature on their abilities to dissolve cellulose.

(1) A low viscosity corresponded to a high conductivity and a high temperature corresponded to a strong ability of the solvent to dissolve cellulose.
(2) The four kinds of DESs were able to dissolve cellulose to varying degrees and the oxalic acid/ChCl DES exerted the best dissolution effects.
(3) Thus, the proposed DES is a ChCl-based solvent, which forms new hydrogen bonds with the hydroxyl groups in the cellulose through an oxygen atom in the hydroxyl group and a nitrogen atom in the amino group. Moreover, after the deep eutectic system formed internal hydrogen bonds, a large number of remaining ions could form hydrogen bonds with the hydroxyl groups of the cellulose, resulting in the strong dissolution of the cellulose. Although cellulose and regenerated cellulose exhibited similar structures, the crystalline form of the cellulose changed from type I to type II.

## Figures and Tables

**Table 1:** The Physical Parameters of deep eutectic solvents (DESs).

DESs	Molar Ratio	Synthesis Temperature (°C)	HBA Melting Point (°C)	HBD Melting Point (°C)	DESs Melting Point (°C)	Water Content (%)	Dissolution Rate at 100 °C (%)	Dissolution Time/h
Urea/ChCl	1:2	80	302	131	12	0.43	1.03	3
Citric acid/ChCl	1:2	80	302	153	37.8	2.65	1.94	2
Oxalic acid/ChCl	1:2	50	302	101	30	20	2.54	1.5
Glycerol/ChCl	1:2	35	302	18.6	−36.5	1.43	0.6	12

**Table 2:** Effect of temperature on dissolution yield of cellulose in oxalic acid/chlorocholine.

Dissolution Temperature (°C)	60	70	80	90	100
**Solubility (%)**	0.50	0.64	0.82	1.96	2.54
